# IgG Fc binding protein (FCGBP) inhibits the development of laryngeal squamous cell carcinoma and cisplatin resistance through the PIGR/JAK2/STAT3 pathway

**DOI:** 10.2478/raon-2026-0003

**Published:** 2026-01-21

**Authors:** Xuemei Wan, Yunlan Zeng, Ming Xiong, Lin Chen, Yundan Bai

**Affiliations:** Department of Otolaryngology Head and Neck Surgery, The Affiliated Hospital of Southwest Medical University, Luzhou, Sichuan, 646000, China; Department of Otolaryngology, Chengdu Integrated TCM & Western Medicine Hospital, Chengdu, Sichuan, 610000, China

**Keywords:** laryngeal squamous cell carcinoma, FCGBP, PIGR/JAK2/STAT3, cisplatin resistance

## Abstract

**Background:**

Laryngeal squamous cell carcinoma (LSCC) is the second most common malignancy of the head and neck, and one of the major therapeutic challenges is resistance to cisplatin (CDDP). IgG Fc binding protein (FCGBP), known as a tumor suppressor in various cancers, has also been implicated in drug resistance. This study investigated the role of FCGBP in LSCC.

**Materials and methods:**

The expression and prognostic relevance of FCGBP were initially analyzed using the Gene Expression Profiling Interactive Analysis 2 (GEPIA2) database. The *in vivo* effects of FCGBP were examined using a nude mouse xenograft model of LSCC, and its *in vitro* effects were assessed through half-maximal inhibitory concentration (IC_50_) analysis, colony formation assays, and flow cytometry. The underlying mechanism by which FCGBP modulates CDDP resistance was invstigated by silencing the polymeric immunoglobulin receptor (PIGR).

**Results:**

FCGBP was significantly downregulated in head and neck squamous cell carcinoma (HNSCC) tissues and LSCC cell lines, and its reduced expression was associated with poor prognosis. It inhibited the viability and proliferation of LSCC cells by approximately 50% and reduced their resistance to CDDP, lowering the IC_50_ from 50 μM to approximately 30 μM. Mechanistically, FCGBP modulated the PIGR/JAK2/STAT3 signaling pathway, thereby exerting both anti-tumor and anti-CDDP resistance effects. *In vivo*, FCGBP overexpression significantly suppressed LSCC tumor growth, with tumor volume reduced by approximately 67%.

**Conclusions:**

These findings suggest that the FCGBP/PIGR/JAK2/STAT3 axis regulates CDDP resistance in LSCC and that FCGBP may serve as a potential therapeutic target to improve cisplatin efficacy in treating LSCC.

## Introduction

Laryngeal squamous cell carcinoma (LSCC) is the second most common malignant tumor of the head and neck, with both incidence and mortality rates increasing annually.^1^ LSCC poses a serious threat to human health and quality of life due to its high rates of local recurrence and metastasis, as well as its poor prognosis.^2^ Cisplatin (CDDP) remains the most commonly used chemotherapeutic agent for LSCC; however, resistance to CDDP develops in over 30% of patients, typically during treatment or within 6 months following CDDP-based chemotherapy.^3,4^ Thus, elucidating the molecular mechanisms underlying CDDP resistance in LSCC, as well as identifying key regulatory factors involved, is essential for the development of effective therapeutic strategies and for improving clinical outcomes.

IgG Fc binding protein (FCGBP), a mucin initially identified in the intestinal epithelium, plays important roles in tumor immunity, metastasis, and the innate defense of mucosal epithelial tissues. Numerous studies have reported that dysregulation of FCGBP is associated with the progression of various cancers.^5,6^ In head and neck squamous cell carcinoma (HNSCC), FCGBP functions as a tumor suppressor gene. For example, upregulation of FCGBP has been shown to significantly inhibit the proliferation and invasion of oral squamous cell carcinoma cells.^7,8^ Additionally, the expression level of FCGBP is correlated with markers of epithelial-mesenchymal transition (EMT) and is associated with the prognosis of patients with HNSCC.^9^ FCGBP has also been identified as a specific biomarker of the immunosuppressive tumor microenvironment and paclitaxel resistance in HNSCC^10^, and its role in drug resistance has been further implicated in colorectal cancer.^11^ Moreover, cinobufacini has been shown to enhance FOXO1-mediated transcription of FCGBP in osteosarcoma, thereby reducing tumor growth and resistance to doxorubicin.^12^ Despite these findings, the role of FCGBP in LSCC and its involvement in resistance to CDDP remain unclear and warrant further investigation.

This study aimed to explore the role of FCGBP in CDDP resistance in LSCC and identify corresponding downstream regulatory pathways, with a particular focus on the polymeric immunoglobulin receptor (PIGR), to identify potential predictive biomarkers and therapeutic targets for overcoming CDDP resistance in LSCC.

## Materials and methods

### Cell culture

Human LSCC cell lines (AMC-HN-8, TU-212, and TU-177), along with normal human bronchial epithelial cells (NHBEC), were obtained from the Shanghai Cell Bank of the Chinese Academy of Sciences. All cell lines were cultured in Dulbecco’s Modified Eagle’s Medium (DMEM; GIBCO), supplemented with 10% fetal bovine serum (FBS; GIBCO) and 1% penicillin-streptomycin. Cells were maintained in a humidified incubator at 37°C with 5% CO_2_.

### Adenovirus-mediated gene silencing and overexpression

Adenoviral vectors were used to achieve either overexpression or silencing of FCGBP and PIGR. Short interfering RNAs (siRNAs) targeting mouse FCGBP (si-FCGBP, 5′-GACCAAAGCTGATCTCATA-3′) and PIGR (si-PIGR, 5′-TCGATCACTCAGGAGACAT-3′) were designed by GenePharma (Shanghai, China). For overexpression, the mouse FCGBP coding sequence was also cloned into an adenoviral vector. An adenoviral empty vector served as the negative control (NC group). Cells were seeded in culture plates and allowed to adhere overnight. Adenovirus infection was performed by replacing the culture medium with serum-free DMEM containing the corresponding adenovirus at a multiplicity of infection (MOI) of 50 plaque forming units (PFU) per cell for 48 hours. The experiment was repeated three times. We used Lipofectamine 3000 (Life Technologies) transfection reagent to transfect siRNA into cells for 48 hours, following the manufacturer’s instructions. Adenoviral infection/transfection efficiency was assessed by western blotting.

### Cell Counting Kit-8 (CCK-8) assay

Cell viability and the half-maximal inhibitory concentration (IC_50_) of CDDP were determined using the CCK-8 kit (Beyotime, Shanghai, China). TU-212 and AMC-HN-8 cells (3,000 cells per well) were seeded in 96-well plates in sextuplicate and treated with a range of CDDP concentrations following adenoviral transfection or non-transfection. After 72 hours, 10 μL of CCK-8 reagent was added to each well and incubated for 2 hours at 37°C. The absorbance at 450 nm was measured using a microplate reader (SpectraMax i3; Molecular Devices, USA). The IC_50_ values were calculated by fitting the dose–response curves using a nonlinear regression model in GraphPad Prism 8.

### Colony formation assay

TU-212 and AMC-HN-8 cells, either transfected or untransfected, were seeded in 6-well plates at a density of approximately 1,000 cells per well. After two weeks of culture, the resulting colonies were fixed with 4% paraformaldehyde and stained with crystal violet. Colony-forming ability was evaluated based on both colony size and density.

### Apoptosis analysis

TU-212 and AMC-HN-8 cells, with or without adenoviral transfection, were seeded in 6-well plates and cultured for 24 hours. The medium was then replaced with serum-free DMEM, and cells were incubated for an additional 24 hours to synchronize the cell cycle in the G_0_/G_1_ phase and minimize background apoptosis due to differences in proliferation. Subsequently, cells were treated with 20 μM CDDP in complete culture medium for 48 hours. Apoptosis was assessed using the fluorescein isothiocyanate (FITC)/Annexin V Apoptosis Detection Kit I (BD PharMingen, CA, USA), following the manufacturer’s instructions. After collection and washing, the cells were stained and analyzed by flow cytometry to determine the apoptosis rate.

### Xenograft formation assay

All animal experiments were approved by the Ethics Committee of Chengdu Integrated Traditional Chinese and Western Medicine Hospital (Approval No. 2024DL-013) and conducted in accordance with the National Institutes of Health Guide for the Care and Use of Laboratory Animals. A total of 24 BALB/c nude mice (4 weeks old) were purchased from the Shanghai Experimental Animal Center (Shanghai, China). Transfected TU-212 cells (1 × 10^7^) suspended in 100 μL of serum-free DMEM were subcutaneously injected into the right flank of each mouse. To minimize bias, tumor cell injections were performed by investigators who were unaware of the group assignments (blinded operator). Mice were then treated with intraperitoneal injections of CDDP (1 mg/kg) or an equivalent volume of saline every two days.^13^ Mice were housed in conventional settings (12-hour day-night cycle, 22 ± 1°C, 50% ± 10% humidity) and had free access to food and water. After one week of acclimatization, mice were randomly assigned to four groups (n = 6) using a random number table: (1) NC, (2) FCGBP, (3) NC+CDDP and (4) FCGBP+CDDP. On day 35, the mice were euthanized, and the tumors were excised, weighed, and collected for further analysis. Tumor collection, weighing, and subsequent immunohistochemistry were also performed by investigators blinded to group identity.

### Immunohistochemical analysis

Tumor tissues were fixed, paraffin-embedded, sectioned, and dried. The sections were incubated with a primary antibody against Ki-67 (1:600 dilution; Abcam, catalog no. ab15580), followed by incubation with an HRP-conjugated goat anti-rabbit IgG H&L secondary antibody (1:20,000 dilution; Abcam, catalog no. ab205718). Immunostained sections were visualized, and representative images were captured using a light microscope (Olympus, Tokyo, Japan).

### Western blot analysis

Western blotting was performed as previously described.^14^ The primary antibodies used were: FCGBP (1:1000; Abcam, ab121202), PIGR (1:1000; Abcam, ab272728), JAK2 (1:1000; Abcam, ab108596), phosphorylated JAK2 (p-JAK2, 1:1000; Abcam, ab32101), STAT3 (1:1000; Abcam, ab68153), phosphorylated STAT3 (p-STAT3, 1:1000; Abcam, ab267373), and GAPDH (1:1000; Abcam, ab9485). HRP-conjugated mouse and goat anti-rabbit secondary antibodies (1:10,000; Abcam) were used for detection.

### Statistical analysis

Statistical analysis was conducted using GraphPad Prism 8 software (GraphPad Software, San Diego, CA, USA). Comparisons between multiple groups were performed using one-way analysis of variance (ANOVA), while comparisons between two groups were analyzed using the two-tailed Student’s t-test. Data are presented as mean ± standard deviation (SD). A *p*-value < 0.05 was considered statistically significant.

## Results

### FCGBP is downregulated in LSCC

**FIGURE 1.**
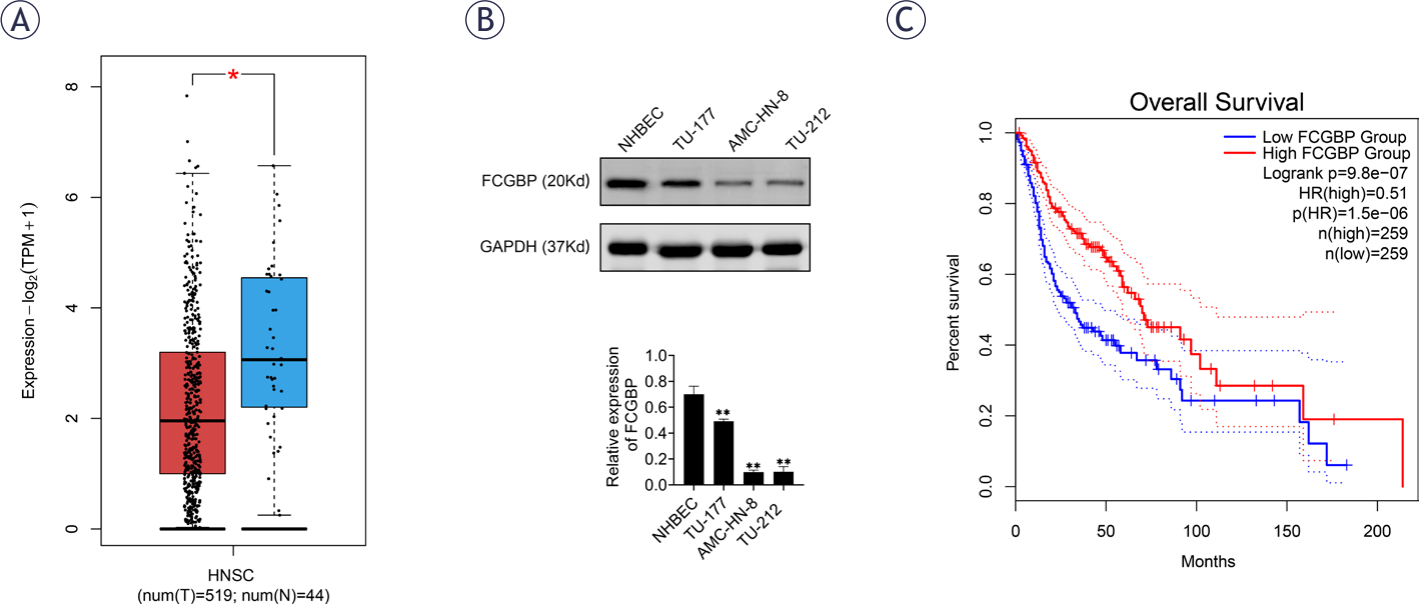
Fc binding protein (FCGBP) is downregulated in laryngeal squamous cell carcinoma (LSCC). **(A)** The Gene Expression Profiling Interactive Analysis 2 (GEPIA2) database showing differential FCGBP expression between head and neck squamous cell carcinoma tissues and normal tissues. **(B)** Western blot analysis of FCGBP protein expression in LSCC cell lines (AMC-HN-8, TU-212, TU-177) and normal human bronchial epithelial cells (NHBEC). **(C)** GEPIA analysis of the association between FCGBP expression and overall survival in LSCC patients. Values are presented as mean ± SD. ** *p* < 0.01 vs. NHBEC; n = 3.

To investigate the expression and potential role of FCGBP in HNSCC, we first analyzed data from The Cancer Genome Atlas (TCGA) using the Gene Expression Profiling Interactive Analysis 2 (GEPIA2) database platform. The results indicated that FCGBP expression was significantly lower in HNSC tissues compared to normal tissues (Figure 1A). To further validate these findings, Western blotting was performed to assess FCGBP expression in normal human bronchial epithelial cells (NHBEC) and three LSCC cell lines (AMC-HN-8, TU-212, and TU-177). FCGBP expression was markedly reduced in all LSCC cell lines relative to NHBEC (Figure 1B). Moreover, Kaplan–Meier analysis revealed that low FCGBP expression was associated with poorer overall survival (Figure 1C). These findings suggest that FCGBP downregulation may be linked to poor prognosis in LSCC; however, additional in vitro and in vivo experiments are required to confirm its tumor suppressor function. FCGBP is generally low-expressed in LSCC tissues, and low expression is associated with poor prognosis. Therefore, low-expression cell lines are closer to the real biological background of LSCC patients and can better simulate the state of FCGBP deficiency or downregulation in the clinic. Therefore, TU-212 and AMC-HN-8 cells were selected for subsequent functional analysis.

### FCGBP suppresses LSCC cell growth

**FIGURE 2.**
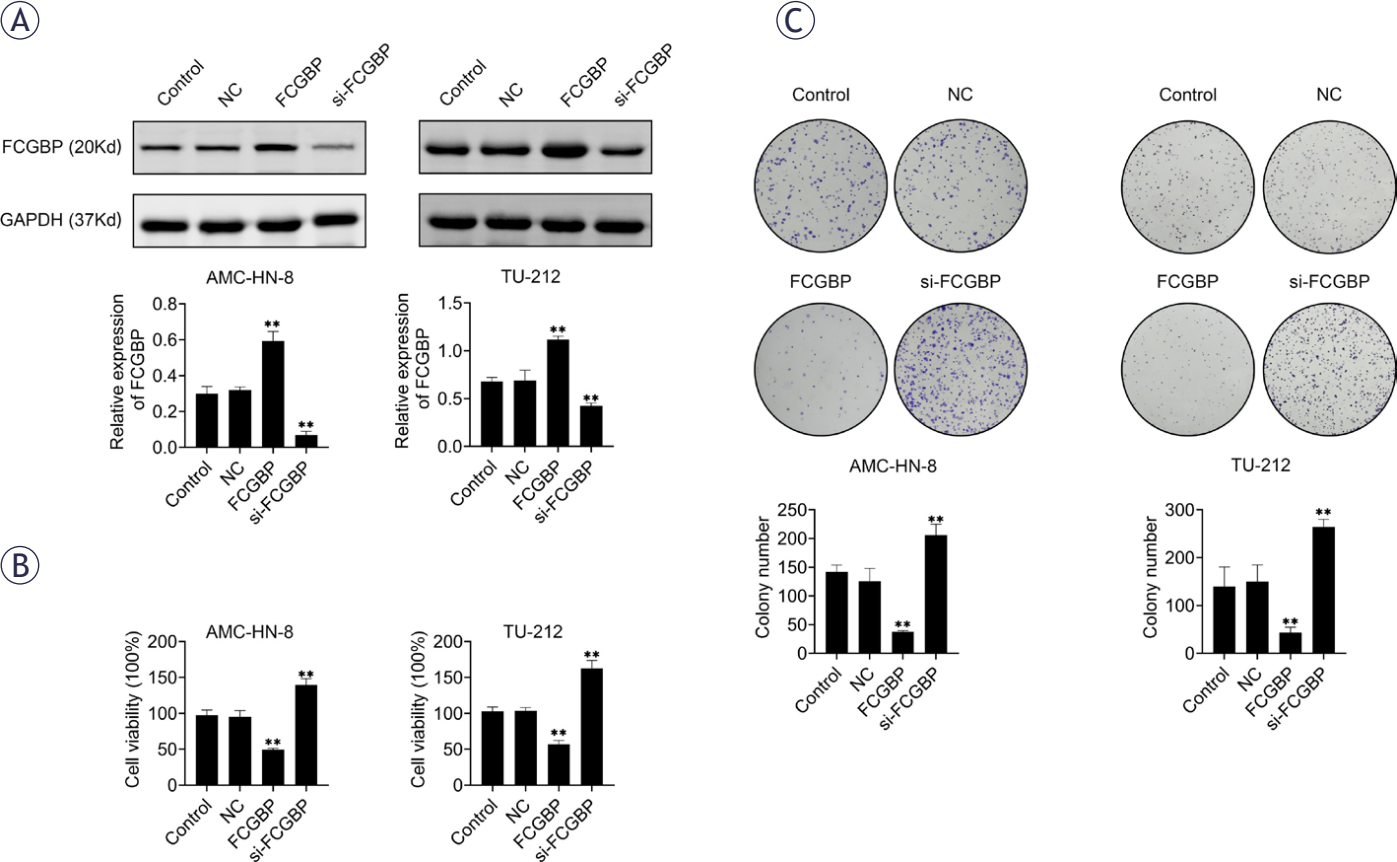
Fc binding protein (FCGBP) inhibits the proliferation of laryngeal squamous cell carcinoma (LSCC) cells. **(A)** Western blot analysis confirming FCGBP overexpression in AMC-HN-8 and TU-212 cells. **(B)** Cell viability measured by Cell Counting Kit-8 (CCK-8) assay. **(C)** Colony formation assessed by crystal violet staining. Values are presented as mean ± SD. ** *p* < 0.01 vs. NC; n = 3.

To elucidate the role of FCGBP in LSCC, we established stable FCGBP-overexpressing and FCGBP-knockdown cell lines (TU-212 and AMC-HN-8) using adenoviral transfection. Successful modulation of FCGBP expression was confirmed by Western blotting (Figure 2A). Functional assays demonstrated that FCGBP overexpression significantly reduced cell viability and colony formation, while FCGBP knockdown exerted the opposite effects (Figures 2C, D). These results indicate that FCGBP plays an inhibitory role in LSCC cell proliferation.

### FCGBP attenuates CDDP resistance in LSCC cells

**FIGURE 3.**
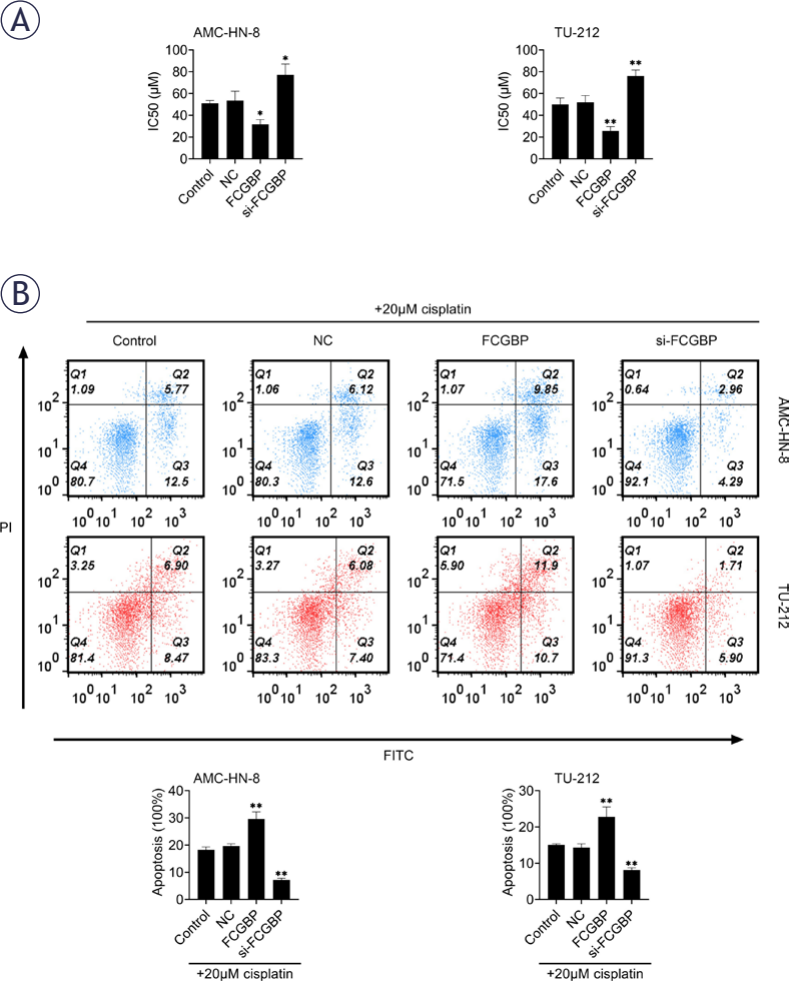
Fc binding protein (FCGBP) reduces cisplatin (CDDP) resistance in laryngeal squamous cell carcinoma (LSCC). **(A)** Cell Counting Kit-8 (CCK-8) assay used to determine the IC50 of CDDP in AMC-HN-8 and TU-212 cells. **(B)** Flow cytometry analysis of apoptosis following treatment with 20 μM CDDP. Values are presented as mean ± SD. * *p* < 0.05, ** *p* < 0.01 vs. NC; n = 3.

Given the established role of FCGBP in chemoresistance across several cancer types, we investigated whether FCGBP modulates CDDP sensitivity in LSCC. Overexpression of FCGBP significantly reduced the IC_50_ value of CDDP in LSCC cells, whereas FCGBP knockdown led to an increased IC_50_ (Figure 3A). Furthermore, FCGBP overexpression enhanced CDDP-induced apoptosis, as evidenced by flow cytometry analysis (Figure 3B). These findings suggest that FCGBP enhances the chemosensitivity of LSCC cells to CDDP.

### FCGBP suppresses LSCC growth and CDDP resistance via the PIGR/JAK2/STAT3 pathway

**FIGURE 4.**
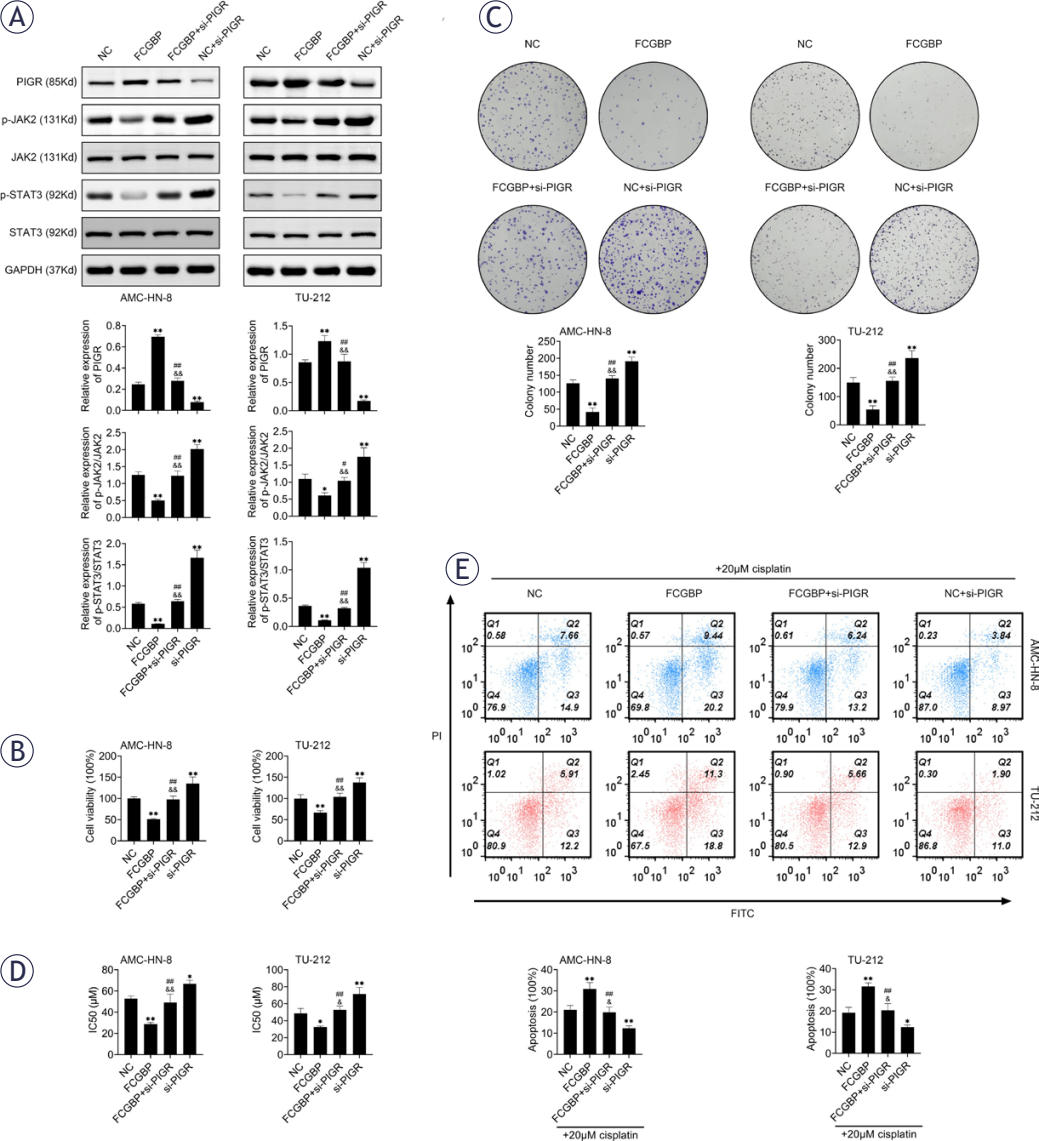
Fc binding protein (FCGBP) inhibits laryngeal squamous cell carcinoma (LSCC) cell growth and cisplatin (CDDP) resistance through the PIGR/JAK2/STAT3 pathway. **(A)** Western blot analysis of PIGR, JAK2, p-JAK2, STAT3, and p-STAT3 protein levels. **(B)** Cell Counting Kit-8 (CCK-8) assay for cell viability. **(C)** Colony formation assessed by crystal violet staining. **(D)** CCK-8 assay for determination of CDDP IC50 in AMC-HN-8 and TU-212 cells. **(E)** Apoptosis detection by flow cytometry in the presence of 20 pM CDDP. Values are presented as mean ± SD. * *p* < 0.05, ** *p* < 0.01 vs NC; ^#^ *p* < 0.05, ^##^ *p* < 0.01 vs FCGBP; ^&^ *p* < 0.05, ^&&^ *p* < 0.01 vs si-PIGR. n=3.

PIGR, a mucin structurally similar to FCGBP, has been reported to exert tumor suppressive effects15,16, whereas the JAK2/STAT3 signaling pathway is known to drive cancer progression.^17,18^ Western blot analysis showed that FCGBP overexpression increased PIGR expression and concurrently inhibited activation of the JAK2/STAT3 pathway (Figure 4A). Importantly, silencing of PIGR reversed the inhibitory effects of FCGBP overexpression on cell proliferation and CDDP resistance (Figures 4B–E), indicating that PIGR is a critical mediator of FCGBP function in LSCC.

### FCGBP inhibits tumor growth and enhances CDDP sensitivity in vivo

**FIGURE 5.**
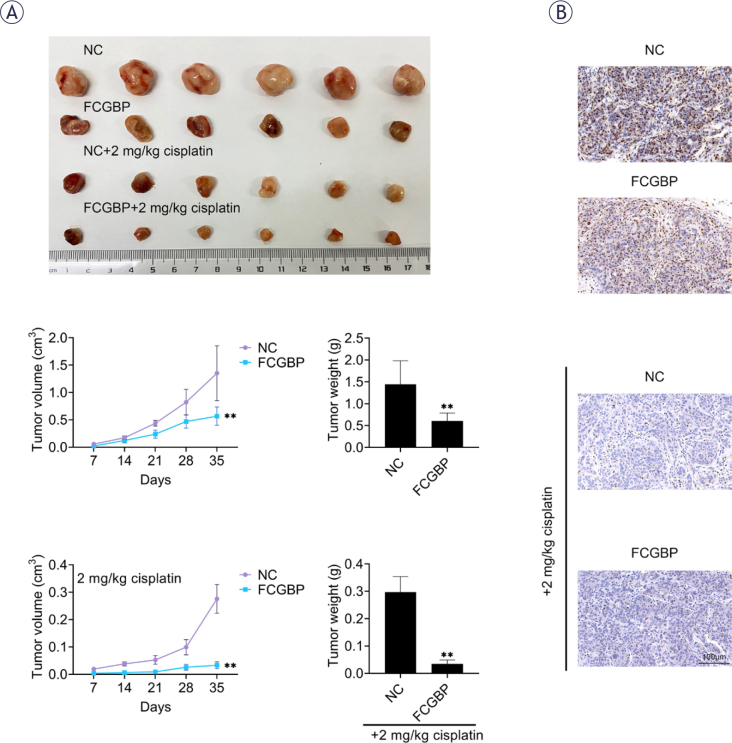
Fc binding protein (FCGBP) suppresses tumor growth and enhances cisplatin (CDDP) sensitivity in vivo. **(A)** Tumor size and weight in xenograft models. **(B)** Immunohistochemical staining of Ki-67 in tumor sections. Values are presented as mean ± SD. ** *p* < 0.01 vs NC. n=6.

To further examine the role of FCGBP *in vivo*, a xenograft mouse model was established using TU-212 cells with or without FCGBP overexpression. In both untreated and CDDP-treated groups, tumors derived from FCGBP-overexpressing cells grew significantly more slowly than those from control cells (Figure 5A). Immunohistochemical analysis revealed reduced Ki-67 expression, a marker of cell proliferation, in tumor sections from FCGBP-overexpressing xenografts in both groups (Figure 5B). These findings confirm that FCGBP inhibits LSCC tumor growth and enhances CDDP sensitivity *in vivo*.

## Discussion

Recurrence and CDDP resistance are the principal causes of treatment failure in patients with LSCC, with the majority of patients developing resistance either during therapy or shortly thereafter, leading to tumor relapse.^19^ A deeper understanding of the mechanisms underlying CDDP resistance may contribute to the development of strategies to overcome chemoresistance and improve clinical outcomes in LSCC. In this study, we demonstrated that FCGBP inhibits tumor cell proliferation both *in vitro* and *in vivo* and enhances the chemosensitivity of LSCC cells to CDDP through modulation of the PIGR/JAK2/STAT3 signaling pathway.

FCGBP is a mucin that has been reported to exhibit low expression across several cancer types, including gallbladder cancer^20^, and has been shown to suppress the initiation and progression of colorectal cancer and HNSCC.^8,21^ Consistent with these observations, previous studies have reported that FCGBP overexpression inhibits the growth of p53 wild-type colorectal cancer cells both in vitro and *in viv*.^22^ Our findings corroborate these reports, showing that FCGBP overexpression similarly inhibits the growth of LSCC cells in both experimental contexts.

CDDP remains one of the most widely used chemotherapeutic agents in clinical practice for treating head and neck cancers and various other solid tumors. However, its therapeutic utility is often limited by adverse effects and the development of drug resistance.^23^ The potential role of FCGBP in modulating drug resistance has garnered increasing attention.^10–12^ In the present study, FCGBP overexpression was found to increase the sensitivity of LSCC cells to CDDP and enhance CDDP-induced apoptosis. Moreover, *in vivo* administration of CDDP in a xenograft mouse model revealed that FCGBP overexpression and CDDP co-treatment synergistically inhibited tumor growth. These findings suggest that FCGBP may serve as both a predictive biomarker for LSCC prognosis and a potential therapeutic target to promote CDDP-induced tumor cell death.

PIGR functions as a transepithelial transporter of polymeric immunoglobulins and plays a vital role in mucosal immunity, sharing functional similarities with FCGBP.^24^ The expression level of PIGR varies across cancer types and has been associated with distinct prognostic outcomes and responses to chemotherapy. In pancreatic cancer, downregulation of PIGR significantly alters cell morphology and diminishes cellular proliferation, adhesion, migration, and invasion.^25^ Furthermore, PIGR expression has been linked to susceptibility and radiosensitivity in patients with HNSCC.^26^ The JAK2/STAT3 signaling pathway, activated by various cytokines, interferons, and growth factors, contributes to numerous physiological and pathological processes, including proliferation, immune responses, inflammation, and cancer development. Dysregulation of this pathway and alterations in related genes are closely associated with immune dysfunction and tumor progression.^18,27^ Gene set enrichment analysis (GSEA) has previously shown that FCGBP is significantly correlated with the JAK2/STAT3 pathway.^28^ However, the regulatory role of FCGBP in modulating the PIGR/JAK2/STAT3 axis has not been well defined. Our results demonstrated that FCGBP upregulates PIGR expression and suppresses JAK2/STAT3 pathway activation, while knockdown of PIGR reverses the inhibitory effects of FCGBP overexpression on LSCC cell proliferation and CDDP resistance, indicating that FCGBP mediates its antitumor and chemosensitizing effects via the PIGR/JAK2/STAT3 pathway.

## Conclusions

In summary, our findings indicate that FCGBP suppresses LSCC tumor growth and reduces CDDP resistance both *in vitro* and *in vivo* by modulating the PIGR/JAK2/STAT3 signaling pathway. These results suggest that FCGBP may serve as a promising therapeutic target to enhance the efficacy of CDDP-based chemotherapy, particularly in patients with CDDP-resistant LSCC.
